# Exploring preference for, and uptake of, rural medical internships, a key issue for supporting rural training pathways

**DOI:** 10.1186/s12913-020-05779-1

**Published:** 2020-10-08

**Authors:** Matthew R. McGrail, Belinda G. O’Sullivan, Deborah J. Russell, Muntasirur Rahman

**Affiliations:** 1 Rural Clinical School, The University of Queensland, Cnr Cambridge & Canning Street, Rockhampton, Queensland 4700 Australia; 2 School of Rural Health, Monash University, PO BOX 666, Bendigo, Victoria 3550 Australia; 3 Rural Clinical School, The University of Queensland, 152 West St, South Toowoomba, Queensland 4350 Australia; 4 Menzies School of Health Research, PO BOX 4066, Alice Springs, Northern Territory 0870 Australia

**Keywords:** Workforce, Rural health services, Junior doctors, Vocational training, Training pathways, Internship

## Abstract

**Background:**

Improved medical care access for rural populations continues to be a major concern. There remains little published evidence about postgraduate rural pathways of junior doctors, which may have strong implications for a long-term skilled rural workforce. This exploratory study describes and compares preferences for, and uptake of, rural internships by new domestic and international graduates of Victorian medical schools during a period of rural internship position expansion.

**Methods:**

We used administrative data of all new Victorian medical graduates’ location preference and accepted location of internship positions for 2013–16. Associations between preferred internship location and accepted internship position were explored including by rurality and year. Moreover, data were stratified between ‘domestic graduates’ (Australian and New Zealand citizens or permanent residents) and ‘international graduates’ (temporary residents who graduated from an Australian university).

**Results:**

Across 2013–16, there were 4562 applicants who filled 3130 internship positions (46% oversubscribed). Domestic graduates filled most (69.7%, 457/656) rural internship positions, but significantly less than metropolitan positions (92.2%, *p* < 0.001). Only 20.1% (551/2737) included a rural location in their top five preferences, less than for international graduates (34.4%, *p* < 0.001). A greater proportion of rural compared with metropolitan interns accepted a position not in their top five preferences (36.1% versus 7.4%, p < 0.001). The proportion nominating a rural location in their preference list increased across 2013–2016.

**Conclusions:**

The preferences for, and uptake of, rural internship positions by domestic graduates is sub-optimal for growing a rural workforce from local graduates. Current actions that have increased the number of rural positions are unlikely to be sufficient as a stand-alone intervention, thus regional areas must rely on international graduates. Strategies are needed to increase the attractiveness of rural internships for domestic students so that more graduates from rural undergraduate medical training are retained rurally. Further research could explore whether the uptake of rural internships is facilitated by aligning these positions with protected opportunities to continue vocational training in regionally-based or metropolitan fellowships. Increased understanding is needed of the factors impacting work location decisions of junior doctors, particularly those with some rural career intent.

## Background

Improved healthcare access for rural populations through a more equitable distribution of the medical workforce remains a major concern internationally [1, 2]. Moreover, addressing health workforce maldistribution is critical to improving health outcome discrepancies in many rural populations [3, 4]. This problem has had extensive policy development at the global scale, however, there is a demand for stronger evidence to inform regional policy makers of effective and well-targeted policies [5, 6].

World Health Organization (WHO) 2010 guidelines recommended a focus on rural education, regulation, financial and professional support, for promoting better healthcare access through improved health workforce supply and retention [7]. These are supported by evidence of the benefit of rural training interventions and policies for improving rural workforce supply and retention [8–10]. This evidence reinforces the positive impact of rural medical school training (the longer the better) and selecting students more likely to practice rurally because of their rural childhood origin or interest in rural work [10–15]. Rural training pathways are critical interventions for producing a skilled, well-distributed and stable rural workforce given that offering financial incentives to shift the established medical workforce into relatively underserved areas have limited effect [16], and are costly [17]. But there remains little published evidence about postgraduate rural pathways which potentially have strong implications for a long-term skilled rural workforce [18–20]. Furthermore, there is only limited information about the uptake of prevocational rural pathways and related internships, in health systems dissimilar to North America’s direct entry pathways to residency training. This paper aims to address this gap by examining uptake and preference of rurally-located internships in one state in Australia.

### Australian policies

Australia uses a broad range of WHO-recommended strategies for rural education, regulation, financial and professional support policies in different forms and with different effects [5]. Regulatory workforce policies, such as those requiring rural return of service in exchange for monetary compensation or awarding visas for overseas trained doctors (OTDs) who work in rural areas, tend to improve rural recruitment but with trade-offs of lower rural retention, especially once the return of service period has been fulfilled, with lower levels of satisfaction having been reported [21–23]. Australia’s key regulatory policy since 1996 has required overseas trained doctors and international students graduating from Australian medical schools to work in Districts of Workforce Shortages (DWS) (mainly in rural areas) for up to 10 years after they enter the medical workforce, in order to gain a provider number for access to Medicare billing. This policy’s ‘success’ is strongly associated with Australia’s current high reliance on OTDs to supply its rural medical workforce [24, 25]. As such, growing the number of domestic graduates who enter and remain in Australia’s rural workforce remains a key priority.

The Australian government has invested in rural medical education policies since the 1990s, with the aim of increasing production of domestic students who choose to work rurally. A key strategy has included funding national Rural Clinical Schools, expanding both existing medical programs and introducing new medical programs at Australian universities that are offered in their entirety within rural areas [5]. These must provide a minimum of 25% of medical students with at least 12 months rural training opportunities and universities must select at least 25% rural background students into their medical schools. This training targets domestic, not international students in most cases [26]. Many doctors have now emerged from this expanded rural training initiative [27], and now increasing attention is being given to post-graduate pathways to retain them in rural areas [28].

To date, rural vocational (specialty) training opportunities have been mainly restricted to general practice (GP). From 2017, the Integrated Rural Training Pipeline (IRTP) strategy aimed to expand other post-graduate training pathways, particularly for graduating students who have trained in Rural Clinical Schools including by creating more rural prevocational and rural registrar training options to attract and keep graduates on a rural training pathway to various specialist career options [29]. Additionally, the IRTP-Specialist Training Program funds 100 new non-GP specialty rural places, where the trainee must spend at least two-thirds of their training in a rural area. The first National Rural Health Commissioner was appointed in 2017 by the Commonwealth government to develop and broker national rural generalist training pathways [30]. New Regional Training Hub managers were also appointed in regions in 2017 to foster medical graduate’s connections to rural pathways [31].

However, supplying new rural vocational positions largely depends on graduate doctors electing to stay in rural prevocational pathways, including during the internship period. In Australia, each doctor’s training pathway is administered by multiple providers including universities (basic qualifications), jurisdictional health services which offer prevocational positions for junior doctors (including newly graduated doctors in their first postgraduate year, hereafter termed ‘interns’) and specialty colleges [32]. Internship training is required for general registration with health services considering interns as the backbone of basic service provision in hospitals [33]. It is a period when new doctors develop broad and generalist skills, get wide exposures to different medical conditions but also begin to confirm career decisions [34]. Rural internships are important for producing a sufficiently distributed and appropriately skilled rural medical workforce [35–37].

In the last decade many Australian states have expanded the number of available rural internship positions. In parallel, there has also been a rapid growth in the numbers of graduating medical students in Australia – around 1425 graduates in 2002 expanding to 3569 in 2016 [32, 38]. This has not been fully matched with growth in internship training places, thus creating angst about securing an intern position which has been evident as national competition for internships increases [39, 40]. Some states, such as Victoria, have used the expansion as an opportunity to grow the proportion of internship positions in rural locations, with a view to improving the geographical maldistribution of its medical workforce. As per Table 1, internship position growth in Victoria across 2010–2017 has seen rural internship positions tripling in number (+ 198%, from 64 to 191), compared to metropolitan areas (+ 28%, from 497 to 634). Australia’s most populated state, New South Wales (NSW), by comparison, saw only modest growth in rural intern positions in a similar period (approximately + 40% in 2011–2016) [35]. Victoria’s overall proportion of rurally located internships rose in 2010–2017 from 11 to 23%, now closely mirroring the state’s rural population [41]. The hope is that this growth is matched with interest and that Australian government investment in rural training for domestic students means that there are sufficient numbers of prospective interns who are both interested in rural work and choose to apply to fill these positions.

**Table 1:** Victoria’s growth of medical graduates and internship positions (metropolitan and rural), 2009–10 to 2016–17^4^

Apply year	Victorian graduates^**3**^	Uptake year	Positions^**1**^	Metropolitan	Large rural (> 50,000)	Smaller rural towns	Combined Rural^**2**^
2009	534	**2010**	561	88.6%	6.2%	5.2%	11.4%
2010	577	**2011**	625	87.8%	6.9%	5.3%	12.2%
2011	721	**2012**	701	83.9%	8.6%	7.6%	16.1%
2012	795	**2013**	705	83.5%	8.4%	8.1%	16.5%
2013	825	**2014**	758	81.1%	10.4%	8.4%	18.9%
2014	742	**2015**	766	78.6%	10.2%	11.2%	21.4%
2015	842	**2016**	813	77.7%	10.3%	11.9%	22.3%
2016	846	**2017**	825	76.8%	10.4%	12.7%	23.2%
Net growth positions (2010–17)			+ 264 (47%)	+ 137 (28%)	+ 51 (146%)	+ 76 (262%)	+ 127 (198%)

^*1*^ *Whilst most graduates take up an internship position in the same jurisdiction as where they graduated, this is not obligated*

^*2*^ *As of 2016, there were 10 metropolitan and 14 rural internship training sites in Victoria*

^*3*^ *Includes all of (a) Domestic Commonwealth supported, (b) Domestic full-fee and (c) International full-fee*

^*4*^ *Source: Postgraduate Medical Council Of Victoria. Annual Reports 2009–10 through to 2016–17*

The objective of this exploratory study, therefore, is to describe and evaluate preferences for, and uptake of, rural internships in Victoria by new Victorian-trained medical graduates (hereafter called *domestic*) and Victorian-trained international medical graduates (*international*) during a period of rural internship position expansion, to inform optimising of rural training pathway policies.

### Internship matching process

Under a formal national agreement of all jurisdictions through the Council of Australian Governments (COAG), Commonwealth supported (subsidised fees) domestic medical graduates are guaranteed to receive an internship position in the same state or territory as they trained. Additionally, Victoria (and some other jurisdictions) extend this guarantee to domestic full fee-paying students (those with Australian or New Zealand citizenship or permanent residency). Competition for remaining internship places is from graduates of Australian medical schools who were international students (full fee-paying students, with temporary residency visas), domestic graduates from schools in other Australian states and territories, and graduates from international medical schools. None of these latter three groups of graduates is guaranteed intern positions in Victoria or any other jurisdiction.

In Victoria, internships are allocated via a statewide centralised process that occurs on a strict timeline [42, 43]. The Postgraduate Medical Council of Victoria (PMCV) administers a matching service on behalf of the Victorian Department of Health and Human Services. Eligibility criteria for participation in Victorian internship matching is detailed in Table 2 and criteria have not changed since they were introduced in 2012/2013. Eligible candidates nominate two professional referees, upload their curriculum vitae, and provide a priority list (ordered preference ranking) of up to 18 health services/internship training programs from a choice of 10 metropolitan internship sites and 14 rural sites as of 2016 [44]. Health services may also request additional information such as a covering letter and may interview candidates in order to produce their lists of preferred candidates. The matching process occurs over three rounds using a computer-based algorithm, which considers each candidate’s preference ranking for internship in different sites and the preferences that health services have for different candidates. No offers of Victorian internship positions are made outside of the statewide PMCV matching process, though if applicants are not computer matched then subsequent direct discussions with a health service may lead to an offer and acceptance. The first round comprises Victorian-trained Australian or New Zealand citizen or permanent resident (‘domestic’) graduates (Priority Group 1). The second round comprises Victorian-trained temporary resident graduates (‘international’) (Priority Group 2). Subsequent offers are made by health services directly to candidates not already matched in either Priority Group 1 or 2, including other groups such as graduates from other Australian jurisdictions or from overseas campus of an accredited Australian or New Zealand medical school.

**Table 2:** Eligibility criteria for different rounds of Victorian Internship Match

Eligible for first round: Victorian-trained domestic graduates (Priority Group 1)	Eligible for second round: Victorian-trained international graduates (Priority Group 2)	Eligible for third round: Graduates from other Australian jurisdictions or from accredited overseas campuses (Priority Group 3)
▪ Australian and New Zealand citizens and Australian permanent residents graduating from a Victorian medical school. Includes both Commonwealth supported and domestic full fee-paying students (i.e. graduates of University of Melbourne, Monash University, Deakin University and University of Notre Dame: Melbourne & Ballarat Clinical Schools).	▪ Australian temporary resident graduates of Victorian medical schools (i.e. international students graduating from the same list of Victorian universities and clinical schools).	▪ Australian and New Zealand citizen/permanent resident graduating from interstate or New Zealand universities (including previous residents of Victoria). ▪ Australian temporary resident graduates of interstate universities; ▪ Graduates from an overseas campus of an Australian/New Zealand University accredited by the Australian Medical Council (e.g. Monash University – Sunway Campus, Malaysia)

## Methods

We used administrative data about all PMCV applicants’ location preferences and accepted locations of Victorian internship positions for 2013–16. These data were de-identified by PMCV and provided to the research team. Data comprised the *preferences* of each candidate (ranking from 1 up to 18); the *internship offer* made to each candidate (either through computerised matching process or directly by a health service following the computerized matching process); the *position matched* from each candidate’s preference list; and the *uptake of each internship* position. Outcomes of interest included preference and uptake of internship positions 2014–17, with internship location geocoded using the Australian standard Modified Monash Model (MMM) rurality scale as MMM-1 ‘metropolitan’; or MMM-2–5 ‘rural’. Additionally, rural locations were stratified as MMM-2 ‘large regional’ (≥50,000 population) and MMM-3-5 ‘smaller regional or rural towns’ (< 50,000 population).

Data were also available about a specific type of internship called the Rural Community-based Internship Training (RCIT) program (now called the Victorian Rural Generalist program). This was introduced in Victoria in 2012, and involved internships based in small rural general practices and hospitals with rotations to larger regional hospitals and community settings. The RCITs are explicitly aimed at improving internship distribution and the number of RCIT positions increased to 35 (4.3%) in 2016 [45], with all five RCIT sites in MMM 3–5 locations.

Covariates of interest included candidate’s Priority Group based on their eligibility (Table 2); candidate’s age category (< 24; 24+ years); and sex (Male; Female).

### Statistical analyses

Descriptive statistics explored the characteristics of candidates relative to their preference list, eligibility group and location of preferred health services/RCIT positions. Data were disaggregated by application year (2013–16), Priority Group (eligibility criteria related to being in first or subsequent rounds of matching process), rurality of internship location preference and position taken, age and gender, before being aggregated for overall statistical testing. Pearson chi-squared tests were used to compare proportions and t-tests to compare means. All calculations were performed using StataSE 15.0 (StataCorp, College Station, TX, USA) and the significance level was α = 0.05.

## Results

### Applicants

Across 2013–16, there were 4562 applicants who filled 3130 internship positions, on average an excess of 358 applications for 782 positions per year (46% oversubscribed). Victorian-trained *domestic graduates* comprised 66.0% (3013/4562) of all applicants, while Victorian-trained *international graduates* comprised 8.1% (368/4562) and the remaining applicants (*other graduates*) comprised 25.9%.

### Preferences

Table 3 reveals that domestic graduates nominated fewer preferences (mean 8.8) compared with international graduates (mean 14.8, *p* < 0.001) and other graduates (mean 11.6, *p* < 0.001). They also had significantly fewer preferences for rural locations (mean 1.7) compared with international graduates (mean 7.1, *p* < 0.001) and other graduates (mean 5.3, *p* < 0.001).

**Table 3:** Priority list, preference matching and internship location of successfully matched applicants (2013–16) for Victorian internship

	Victorian-trained domestic graduates (***n*** = 2737) (Priority Group 1)		Victorian-trained international graduates (***n*** = 262) (Priority Group 2)		Other graduates (***n*** = 131) (Priority Group 3)	Total interns (***n*** = 3130)
	Computer matched (***n*** = 2592)	Through discussion (***n*** = 145)	Computer matched (***n*** = 179)	Through discussion (***n*** = 83)	Through discussion (***n*** = 131)	Overall
**Interns’ preference list for hospital/health service**						
Total number of preferences: mean (SD)	8.8 (2.9)	8.6 (3.7)	15.0 (4.9)	14.3 (4.5)	11.6 (6.6)	9.4 (3.8)
Total preferences for metropolitan locations: mean (SD)	7.1 (2.2)	6.7 (2.5)	7.8 (2.2)	7.4 (2.4)	6.3 (3.3)	7.1 (2.3)
Total preferences for rural locations: mean (SD)	1.7 (2.1)	1.8 (2.3)	7.2 (4.1)	6.9 (4.2)	5.3 (4.8)	2.3 (3.0)
**Proportion of rural (MMM 2–5) location preferences as percentage of total preferences**						
0%	31.1% (807)	32.4% (47)	5.6% (10)	4.8% (4)	21.4% (28)	28.6% (896)
1–20%	37.5% (971)	33.8% (49)	7.3% (13)	9.6% (8)	13.0% (17)	33.8% (1058)
21–50%	25.4% (657)	28.3% (41)	44.7% (80)	47.0% (39)	24.4% (32)	27.1% (849)
51–100%	6.1% (157)	5.5% (8)	42.5% (76)	38.6% (32)	41.2% (54)	10.5% (327)
**Any rural (MMM 2–5) location in top five (5) preferences?**						
Yes	20.1% (522)	20.0% (29)	35.2% (63)	32.5% (27)	48.9% (64)	22.5% (705)
No	79.9% (2070)	80.0% (116)	64.8% (116)	67.5% (56)	51.1% (67)	77.5% (2425)
**Any smaller regional or rural town (MMM 3–5) location in top five (5) preferences?**						
Yes	8.2% (212)	7.6% (11)	22.9% (41)	18.1% (15)	32.1% (42)	10.3% (321)
No	91.8% (2380)	92.4% (134)	77.1% (138)	81.9% (68)	67.9% (89)	89.7% (2809)
**Candidate’s preference that was matched**						
1st preference	51.2% (1327)		26.3% (47)			43.9% (1374)
2nd – 3rd preference	25.0% (649)		16.2% (29)			21.7% (678)
4th – 5th preference	13.7% (355)		15.6% (28)			12.2% (383)
6th or higher preference	10.1% (261)		41.9% (75)			10.7% (336)
Not applicable/ no information	–	145	–	83	131	11.5% (359)
**Accepted internship location**						
Metropolitan (MMM-1)	86.3% (2236)	30.3% (44)	53.1% (95)	47.0% (39)	45.8% (60)	79.0% (2474)
Large regional (MMM-2)	7.8% (203)	19.3% (28)	18.4% (33)	14.5% (12)	22.9% (30)	9.8% (306)
Smaller regional or rural towns (MMM −3-5)^a^	5.9% (153)	50.3% (73)	28.5% (51)	38.5% (32)	31.3% (41)	11.2% (350)
^a^**RCIT** positions	62	20	18	13	15	128

*SD* Standard deviation; *MMM* Modified Monash Model (rurality scale); *RCIT* Rural community internship training; Priority Group = 3 levels as per definitions in Table 2

^a^ Smaller regional or rural towns (MMM 3–5) includes RCIT positions

Of those accepting positions, 31.2% (854/2737) of domestic graduates did not have any rural locations in their preference list. Only 20.1% (551/2737) had at least one rural location in their top five preferences, which was a significantly lower proportion than for international graduates (34.4%, *p* < 0.001) and other graduates (48.9%, *p* < 0.001).

### Matching and uptake

Across 2013–16, the distribution of available internship positions by MMM was *n* = 2474 (79.0%); *n* = 306 (9.8%) and *n* = 350 (11.2%) (for MMM-1, MMM-2 and MMM 3–5 respectively). MMM 3–5 included 128 RCIT positions. Overall, 89.9% of domestic graduates matched with one of their top five preferences, compared with 58.1% (*p* < 0.001) of international graduates. Additionally, 83.3% of domestic graduates (*n* = 2280/2737) took up metropolitan positions (Table 3).

A greater proportion of rural interns than metropolitan interns matched a preference not in their top five choices (36.1% vs. 7.4%, *p* < 0.001) and were aged 24+ years (85.8% vs. 77.3%, *p* < 0.001) (Table 4). A smaller proportion of rural than metropolitan interns were domestic graduates (69.7% vs. 92.2%, *p* < 0.001). There was no difference in the proportion of female interns in metropolitan and rural locations.

**Table 4:** Interns’ matched preferences and characteristics relative to internship location

	Accepted metropolitan (MMM-1) positions (*n* = 2474)	Accepted rural (MMM 2–5) position (*n* = 656)	Accepted large regional (MMM-2) position (*n* = 306)^a^	Accepted non- RCIT smaller regional or rural town (MMM 3–5) position (*n* = 222)^a^	Accepted RCIT position (*n* = 128)^a^
**Preference that was matched**					
Match 1st preference	51.0% (1262)	24.5% (161)	29.7% (91)	11.7% (26)	34.4% (44)
Match: 2nd-3rd preference	25.9% (641)	10.7% (70)	14.7% (45)	4.5% (10)	11.7% (15)
Match: 4th – 5th preference	14.2% (350)	8.8% (58)	11.1% (34)	8.6% (19)	3.9% (5)
Match: outside top five preferences	7.4% (184)	36.1% (237)	30.7% (94)	49.6% (110)	25.8% (33)
Non-algorithm match/NA	1.5% (37)	19.8% (130)	13.7% (42)	25.7% (57)	24.2% (31)
**Age**					
24+ years	77.3% (1912)	85.8% (563)	83.3% (255)	86.0% (191)	91.4% (117)
< 24 years	22.7% (562)	14.2% (93)	16.7% (51)	14.0% (31)	8.6% (11)
**Gender**					
Female	53.6% (1325)	50.2% (329)	54.9% (168)	42.3% (94)	52.3% (67)
Male	46.4% (1149)	49.9% (327)	45.1% (138)	57.7% (128)	47.7% (61)
**Eligibility (priority) group**					
Group 1	92.2% (2280)	69.7% (457)	75.5% (231)	64.9% (144)	64.1% (82)
Group 2	5.4% (134)	19.5% (128)	14.7% (45)	23.4% (52)	24.2% (31)
Group 3	2.4% (60)	10.8% (71)	9.8% (30)	11.7% (26)	11.7% (15)

*MMM* Modified Monash Model (rurality scale); *RCIT* Rural community internship training; Priority Group = 3 levels as per definitions in Table 2

^a^ Any rural (MMM2–5) = Large regional + smaller regional / rural town + RCIT (smaller rural)

Overall, in 2013–2016, only 189 internship positions offered through the matching process were declined, mostly (98%) by domestic graduates. Most declined offers were in metropolitan locations (125, 66.1%), though proportionally rural positions were 2.7 times more likely to be declined compared with metropolitan positions. Among the 64 declined rural offers, 14 (21.9%) had matched their 1st preference, 19 (29.7%) 2nd – 5th preference and 31 (48.4%) 6th or lower preference positions. Most declined metropolitan internship offers (91.2%) were amongst a candidate’s top five preferences, significantly higher than for declined rural offers (51.6%, *p* < 0.001).

### Preference patterns over time

Table 5 shows the preferences and uptake of internships over each of 4 years. Between 2013 and 2016, there was a significant increase in the average number of preferences listed per candidate (from 8.0 to 9.9, *p* < 0.001), for rural positions (from 1.4 to 2.7, *p* < 0.001) and matching RCIT positions nominated as a top five preference (from 1.2 to 3.2%, *p* = 0.013). The proportion of interns not including any rural location in their preference list also decreased significantly overall (from 44.1 to 18.8%, *p* < 0.001).

**Table 5:** Trends in preferences amongst those accepting internships for computer-matched interns, by calendar year 2013–16

Application year	2013	2014	2015	2016	Overall
**Matched metropolitan interns**					
Metropolitan locations filled by matching process % (n)	**(*n*** **= 565)**	**(*n*** **= 583)**	**(*n*** **= 590)**	**(*n*** **= 593)**	**(*n*** **= 2331)**
Match 1st preference	52.4% (296)	55.2% (322)	50.7% (299)	51.6% (306)	52.5% (1223)
Match: 2nd-3rd preference	31.3% (177)	23.8% (139)	26.4% (156)	25.0% (148)	26.6% (620)
Match: 4th – 5th preference	11.9% (67)	13.7% (80)	14.9% (88)	16.5% (98)	14.3% (333)
Match: outside top five preferences	4.4% (25)	7.2% (42)	8.0% (47)	6.9% (41)	6.7% (155)
**Matched regional/rural interns**					
Regional/rural filled by matching % (n)	**(*n*** **= 106)**	**(*n*** **= 102)**	**(*n*** **= 95)**	**(*n*** **= 137)**	**(*n*** **= 440)**
Match 1st preference	32.1% (34)	18.6% (19)	48.4% (46)	38.0% (52)	34.3% (151)
Match: 2nd-3rd preference	13.2% (14)	17.7% (18)	11.6% (11)	11.0% (15)	13.2% (58)
Match: 4th – 5th preference	18.9% (20)	11.8% (12)	9.5% (9)	6.6% (9)	11.4% (50)
Match: outside top five preferences	35.9% (38)	52.0% (53)	30.5% (29)	44.5% (61)	41.1% (181)
**Matched (and accepted) preferences**	**(*n*** **= 671)**	**(*n*** **= 685)**	**(*n*** **= 685)**	**(*n*** **= 730)**	**(*n*** **= 2771)**
Mean # preferences: Total (SD)	8.0 (±3.2)	9.6 (±3.9)	9.3 (±2.6)	9.9 (±3.7)	9.2 (±3.5)
Mean # preferences: regional/ rural (SD)	1.4 (±2.2)	2.2 (±3.1)	1.7 (±1.8)	2.8 (±3.1)	2.0 (±2.7)
% with no regional/rural locations in preference list	44.4% (298)	30.2% (207)	25.6% (175)	18.8% (137)	29.5% (817)
% with any rural (MMM 2+) in top five	25.2% (169)	16.6% (114)	17.4% (119)	25.1% (183)	21.1% (585)
% with MMM 3–5 in top five	7.6% (51)	8.9% (61)	7.3% (50)	12.5% (91)	9.1% (253)
% matching RCIT position in top five	1.2% (8)	2.3% (16)	2.3% (16)	3.2% (23)	2.3% (63)

*SD* Standard deviation; *MMM* Modified Monash Model (rurality scale); *RCIT* Rural community internship training

## Discussion

Our study is the first to explore patterns of internship preferences and uptake in Australia across an entire jurisdiction over time. Our results suggest that domestic graduates are under-represented in rural positions, filling 70% of those positions compared with 92% of metropolitan positions. Also, over half of the rural positions are filled by applicants who either did not prioritise a rural internship position in their top five preferences or were matched outside of the computer algorithm. These findings suggest that a majority of domestic graduates may not be attracted to rural internships, and those doing rural internships tended to not have selected their internship location as a high preference. As such, it appears that rural internships may not be attractive to sufficient numbers of this cohort, and that expanding the numbers of rural internship positions alone may not effectively enhance domestic rural workforce supply. Policy is needed which addresses how to attract greater numbers of domestic students to opt for rural internships, especially those emerging from undergraduate training in Rural Clinical Schools, as this cohort is likely to have the most experience of and interest in rural medicine.

Evidence of specific reasons why rural internships are less attractive is weak. A recent study of interns in another Australian state (New South Wales) using qualitative methods and focus group discussions suggested that rural internships provided high quality training and supervisory support and increased clinical work opportunities, mostly undertaken as part of a smaller team [46]. However, many feared this may limit their exposure to higher acuity presentations, restrict research opportunities and remove networking opportunities, potentially disadvantaging future applications to specialty training. Additionally, high levels of competition for vocational training positions from increased graduate numbers may perpetuate the belief of many rural interested candidates that establishing professional connections with clinical directors of large metropolitan units is critical in order to gain entry into specialty training [32, 47]. A recent opinion piece from Australia’s rural medical students reiterates fears of reduced access to training programs and missing out on key professional relationships and supports via rural internships [48]. A national study reinforced this, showing metropolitan junior doctors were more satisfied with the network of doctors supporting them than rural junior doctors although the latter had higher overall satisfaction and were significantly more satisfied with work-life balance, ability to obtain leave, study time and access to leisure interests than metropolitan counterparts [49].

Given this context, it is likely that many junior doctors – even those with a strong interest in rural practice – feel pressured to choose to train in metropolitan locations. As such, increased uptake of rural internships may be facilitated by aligning these positions with protected opportunities to access vocational training in regionally-based or metropolitan fellowships, with a key option being weighted selection criteria to specialist colleges for doing internships in regional locations. Many qualities of rural intern positions have good potential to attract domestic students, should common concerns related to professional networks and accessing sought after vocational training pathways be adequately addressed [50]. Future research could examine the nature of professional networks needed by rural junior doctors for career progression in different specialties and what interventions might be most effective. Addressing such research gaps may assist rural policy makers and health services to devise better strategies to attract students hoping to become rural specialists, giving them more confidence or certainty that a rural internship will connect with ongoing rural specialty training opportunities [51]. This is particularly important as this very early stage of medical graduates’ careers (i.e. internship year) is a key time for ‘settling down’ in a location and therefore comprises an additional opportunity to increase rural immersion and positive role models. This may reduce the risk of “urban narcissism” towards rural doctors [52], increase their interest in generalist careers that fit with most rural locations [53], and strengthen their connections to specific regions where they have previously trained and lived [54].

Currently, specialty college entry criteria tend to prioritise the achievement of focused skills, rather than broader experience that is typical of medical practice in rural areas, despite diluted learning acknowledged in crowded metropolitan tertiary hospital training environments [35]. This focus is increasingly seeing medical graduates pursue specific technical skills including pursuing research publications, which may be more challenging to achieve in rural locations [55]. A recent snapshot survey of the broad intentions of the next generation of rural doctors (current undergraduates attending a rural conference) suggested they had rural generalist career intentions but were wedded to doing part of their postgraduate training in metropolitan areas [56]. Linking this group with rural internship positions (and beyond) as part of a continuous and secure regional postgraduate training programs with a line of sight to specialist college selection, may be important to increase the attractiveness of rural internships for this group, but this needs to be clarified with further research.

Our findings may also have important implications for policies concerning how to prepare international graduates of Australian medical schools. Currently (notwithstanding the effect of COVID-19), universities are heavily reliant on income generated from these students, which subsidise training of domestic graduates. Come the intern year, however, the tensions between producing a self-sufficient, well-distributed domestic medical workforce (including retaining graduates from rural undergraduate clinical training) and providing international graduates with requisite internships in order to gain general medical registration becomes apparent. Approximately 70% of this group stays in Australia and secure an internship, but universities are keen to maximise internship opportunities for international graduates so that the medical degrees that they offer remain attractive. Intern placement prioritisation for international graduates differs between jurisdictions. In Victoria, they are prioritised higher than all medical graduates from other jurisdictions, such as those who may have grown up in rural Victoria but travelled to a different state for their medical training. The latter example are perhaps more likely to work long-term in rural Victoria if given the opportunity to return to Victoria and take up a rural internship. International graduates in comparison are less likely to have any connection to rural areas they are applying to undertake an internship in, as the government-funded rural training opportunities during medical school (Rural Clinical School training immersion) is largely reserved for domestic students [25, 26]. They are also unlikely to have spent childhood periods in rural Victoria. This lack of connection to place is expected to reduce recruitment to rural practice and longer term retention [54]. Opportunities remain, therefore, for selection processes to consider implications for medical workforce distribution ahead of implications for Victoria’s international market for medical education. In NSW, for example, domestic medical students graduating from other jurisdictions are prioritised ahead of international students.

Our study has some limitations largely related to its use of administrative data. For example, it was unable to link preference and uptake outcomes with two key factors associated with rural practice, namely, having a rural background and prior rural training during basic medical training. Other potentially important data items not available were career intent, particularly for generalist versus specialist choice, as well as whether individuals were required to serve any rural bonded periods. This may be relevant to explore whether including the doctor’s interests to match against longer-term regional workforce planning imperatives as part of selecting for rural internships improves uptake. This may achieve a better match of candidates for the region, over the current computer-generated match that is relatively impersonal. Career decision making of junior doctors is known to be complex and multifactorial, with considerations including place preferences, partner needs and training demands overlaid on the doctor’s work expectations related to their career and broader life [57–59]. Despite the limitations of using administrative data, these allowed us to capture the whole Victorian cohort over time, thereby avoiding issues of non-response bias. A further limitation is that the study setting is a single Australian jurisdiction, and internship policies differ by jurisdiction. Our study was additionally not able to link the internship location with longer-term work location outcomes, particularly relating to career specialty, although this is planned as part of further work. Finally, this study did not capture the reasons or motivations behind each candidate’s preferencing and uptake decisions nor what strategies may be effective to increase both preferencing and uptake of rural internships by domestic graduates, particularly those emerging from rural undergraduate training. These elements could be explored in further research.

## Conclusions

The finding that most rural positions tend to be filled by applicants either matched outside of their top five preferences or by unmatched candidates and international graduates indicates that the expanded number of rural internships in Victoria are not sufficiently attractive to applicants, particularly to domestic graduates. The findings suggest that it may be important to focus on strategies to promote greater uptake of rural internships by domestic graduates (particularly retaining graduates from rural undergraduate medical training). This may rely on Rural Clinical Schools and Regional Training Hubs working with regional health services, students, clinicians and specialty colleges to address the attractiveness of rural internships to domestic graduates. Further research could explore whether the uptake of rural internships is facilitated by aligning these positions with protected opportunities to continue vocational training in regionally-based or metropolitan fellowships. Moreover, increased understanding is required of the factors impacting work location decisions of junior doctors, particularly those with some rural career intent.

## Data Availability

The datasets produced and/or analysed during the current study are available from the corresponding author on reasonable request.

## Abbreviations

COAG: Council of Australian governments
GP: General practice
IRTP: Integrated rural training pipeline
MABEL: Medicine in Australia: balancing employment and life (study)
MMM: Modified monash model (rurality scale)
NOSM: Northern Ontario school of medicine
NSW: New South Wales
OTD: Overseas trained doctor
PMCV: Postgraduate medical council of Victoria
RCIT: Rural community internship training
WHO: World health organization
